# Melorheostosis of the Hip: A Case Report

**DOI:** 10.7759/cureus.94263

**Published:** 2025-10-10

**Authors:** Shuichi Fujiwara, Ikuo Fujita, Toshiyuki Takemori, Takuya Fujimoto, Shunsuke Yahiro, Ami Sawada, Ryosuke Kuroda

**Affiliations:** 1 Department of Orthopaedic Surgery, Hyogo Cancer Center, Akashi, JPN; 2 Department of Orthopaedic Surgery, Kobe University Graduate School of Medicine, Kobe, JPN

**Keywords:** hyperostosis, leri’s disease, manipulation, melorheostosis, surgical intervention

## Abstract

Melorheostosis, also known as Leri’s disease, is a rare sclerosing bone dysplasia characterized by abnormal overgrowth of cortical bone, typically appearing as “dripping candle wax” on imaging. It most commonly affects the diaphyseal regions of the long bones in the lower limbs and may result in limb-length discrepancy, joint stiffness, progressive deformity, and soft tissue ossification. Owing to its rarity, treatment approaches are usually individualized. Here, we report the case of a 41-year-old female who presented with progressive limitation of right hip mobility since childhood, which began to interfere with daily activities. Imaging revealed extensive soft tissue ossification and endomedullary sclerosis around the right hip with cortical excrescence resembling candle wax dripping, suggesting melorheostosis. Following multidisciplinary evaluation, surgical excision of the bony mass and passive manipulation of the hip joint were performed. Histological examination confirmed a dense, compact bone resembling cortical bone. Postoperatively, hip flexion improved from 20° to 60° and abduction improved from 15° to 20°, with no evidence of joint instability. At the 30-month follow-up, mild limitation of motion persisted (flexion, 45°; abduction, 25°); however, the patient maintained good function with no significant impact on daily activities.

## Introduction

Melorheostosis, also referred to as Leri’s disease, is a rare skeletal disorder characterized by areas of excessive cortical bone formation, producing a characteristic appearance on imaging [1,2]. The term originates from the Greek words meaning limb (melos) and flow (rhéō), reflecting the condition’s hallmark flowing pattern of bone overgrowth [3]. Its prevalence is estimated at approximately 0.9 cases per million individuals, with an incidence of roughly one in a million. This condition affects males and females equally and can occur in both pediatric and adult populations [4]. It predominantly involves the diaphyseal regions of the long bones in the lower extremities, with uncommon axial skeletal involvement. It may be associated with clinical manifestations, such as limb length inequality, restricted joint mobility, progressive skeletal deformity, and ectopic ossification in the surrounding soft tissues [1]. Patients with lower extremity involvement exhibited marked functional impairments, often necessitating the use of assistive devices due to substantial reductions in mobility. Although upper extremity lesions were less prevalent, they were nonetheless associated with moderate limitations, especially in activities requiring fine motor dexterity [5].

In adults, melorheostosis typically presents with four distinct radiographic patterns on plain radiographs: the classic “dripping candle wax” appearance, osteoma-like lesions, myositis ossificans-like lesions, and osteopathia striata-like lesions. In some cases, a mixed radiographic presentation may be observed. The differential diagnosis of melorheostosis includes a variety of conditions that may present with overlapping clinical or radiographic features, such as osteoma, parosteal osteosarcoma, localized scleroderma, synovial osteochondromatosis, and Caffey disease [6]. Although diagnosis is usually based on characteristic imaging features, histopathological evaluation may be necessary when radiographic findings are atypical or inconclusive. Histologically, melorheostotic lesions often show increased cortical bone density, disorganized woven bone, prominent osteoid deposition, and enhanced vascularity [7].

Melorheostosis is managed with both conservative and surgical treatments aimed at pain relief and improving quality of life. Conservative methods include bisphosphonates, physiotherapy, bracing, casting, sympathectomy, and nerve blocks. Surgery, such as tendon lengthening or bone excision, is considered when these are ineffective [5,8]. Surgical complications include not only recurrence after excision but also soft tissue contracture in cases with significant soft tissue involvement, with some cases requiring joint replacement [9].

## Case presentation

A 41-year-old female was referred to the Hyogo Cancer Center (Akashi City, Hyogo, Japan) with limited joint mobility of the right hip. Since elementary school, she had difficulty moving the right hip joint and had been under observation at a nearby clinic. At the age of 30 years, calcification was noted in the right hip; however, owing to mild symptoms, observation was continued. Recently, owing to the limited range of motion of the right hip joint, the patient was referred to our hospital for further examination and treatment. The range of motion of the right hip on admission was as follows: flexion, 20°, and abduction, 15°.

**Figure 1:**
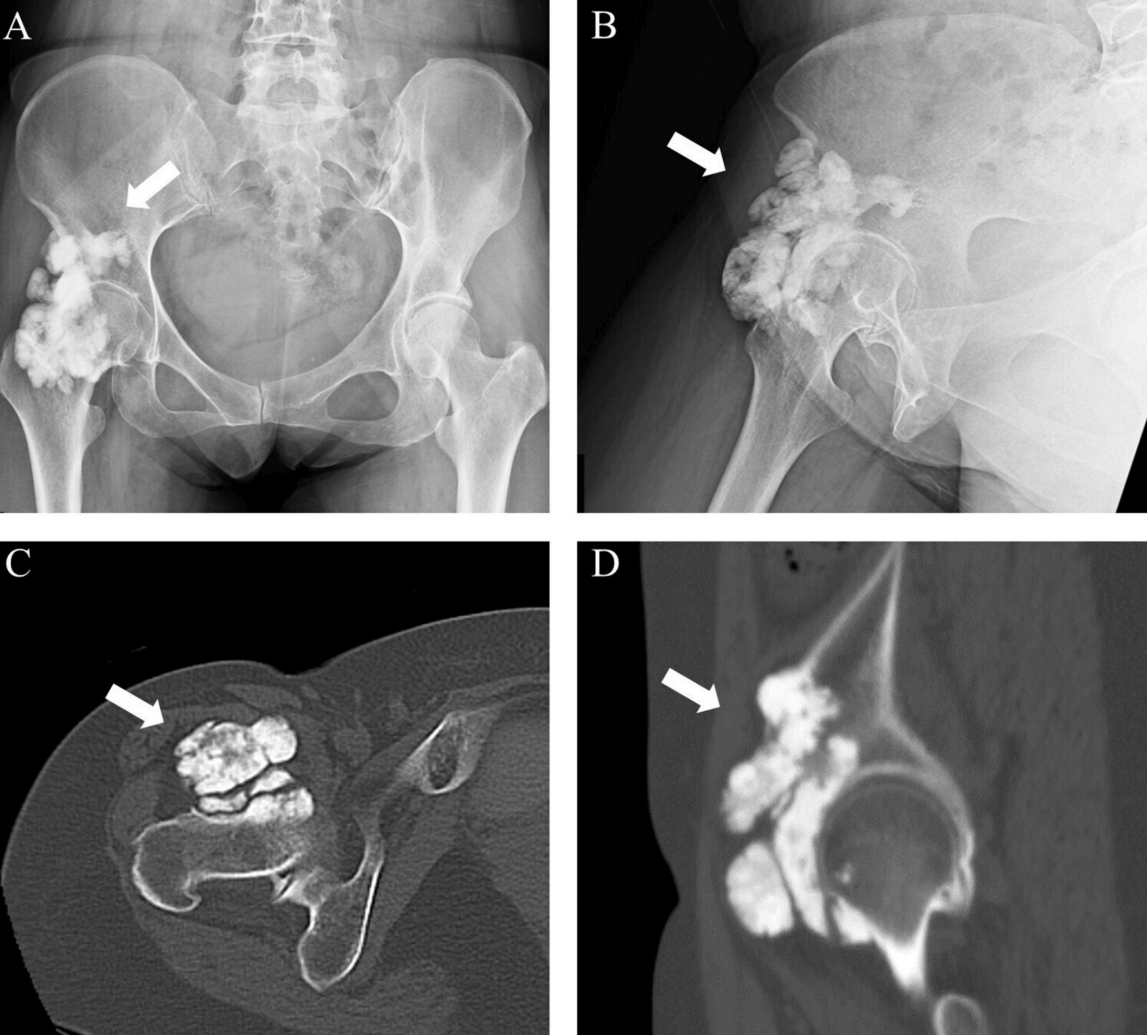
Plain radiographs of the right hip in anteroposterior position (A) and lateral position (B). Computed tomography (CT) of the right hip in the axial (C) and sagittal (D) views. The white arrow indicates the position of the mass. The mass was located anterior to the hip joint.

**Figure 2:**
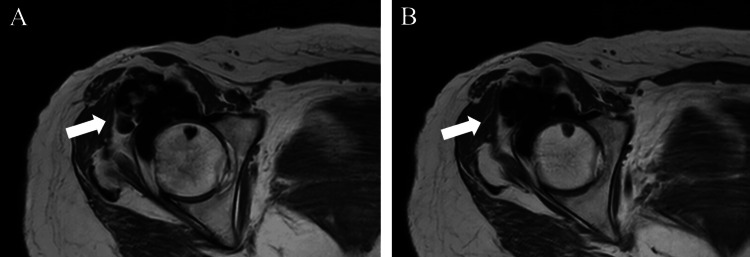
T1-weighted (A) and T2-weighted (B) images of the mass (white arrow) on magnetic resonance imaging (MRI) of the right hip.

Plain radiography revealed extensive soft tissue ossification around the right hip (Figure 1). Computed tomography (CT) confirmed endomedullary sclerosis abutting the cortex with subtle excrescence in front of the femoral head and neck, resembling candle wax dripping (Figure 1). Magnetic resonance imaging (MRI) revealed low signal intensities of the mass on T1-weighted (Figure 2A) and T2-weighted images (Figure 2B).

Melorheostosis was diagnosed based on clinical course and imaging findings. As hip mobility gradually worsened and began to affect her daily life, she opted for surgical treatment to improve her range of motion and activities of daily living. The risks of postoperative recurrence and possible neurological complications were explained prior to the surgery, and informed consent was obtained.

**Figure 3:**
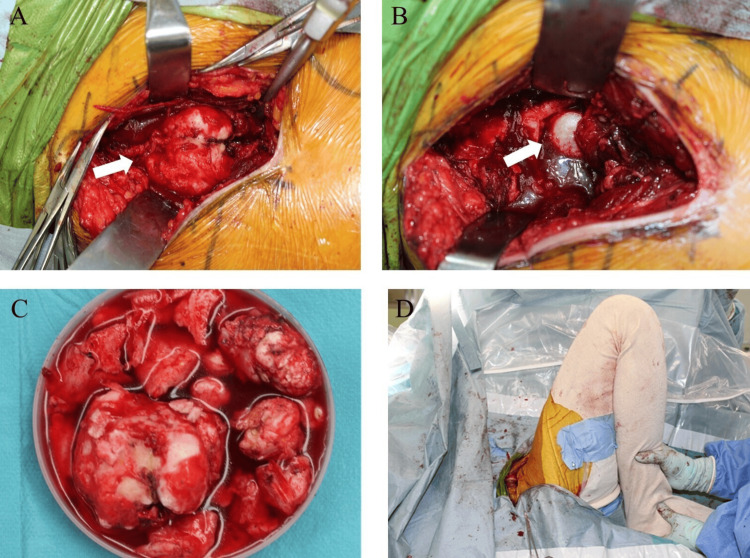
Intraoperative photographs. (A) Bony mass (white arrow) is detected in the deep region underlying the rectus femoris muscle. (B) The femoral head (white arrow) is exposed following excision of the mass and partial resection of the joint capsule. (C) The mass causing impingement is resected as much as possible. (D) Hip joint manipulation is performed after mass resection.

**Figure 4:**
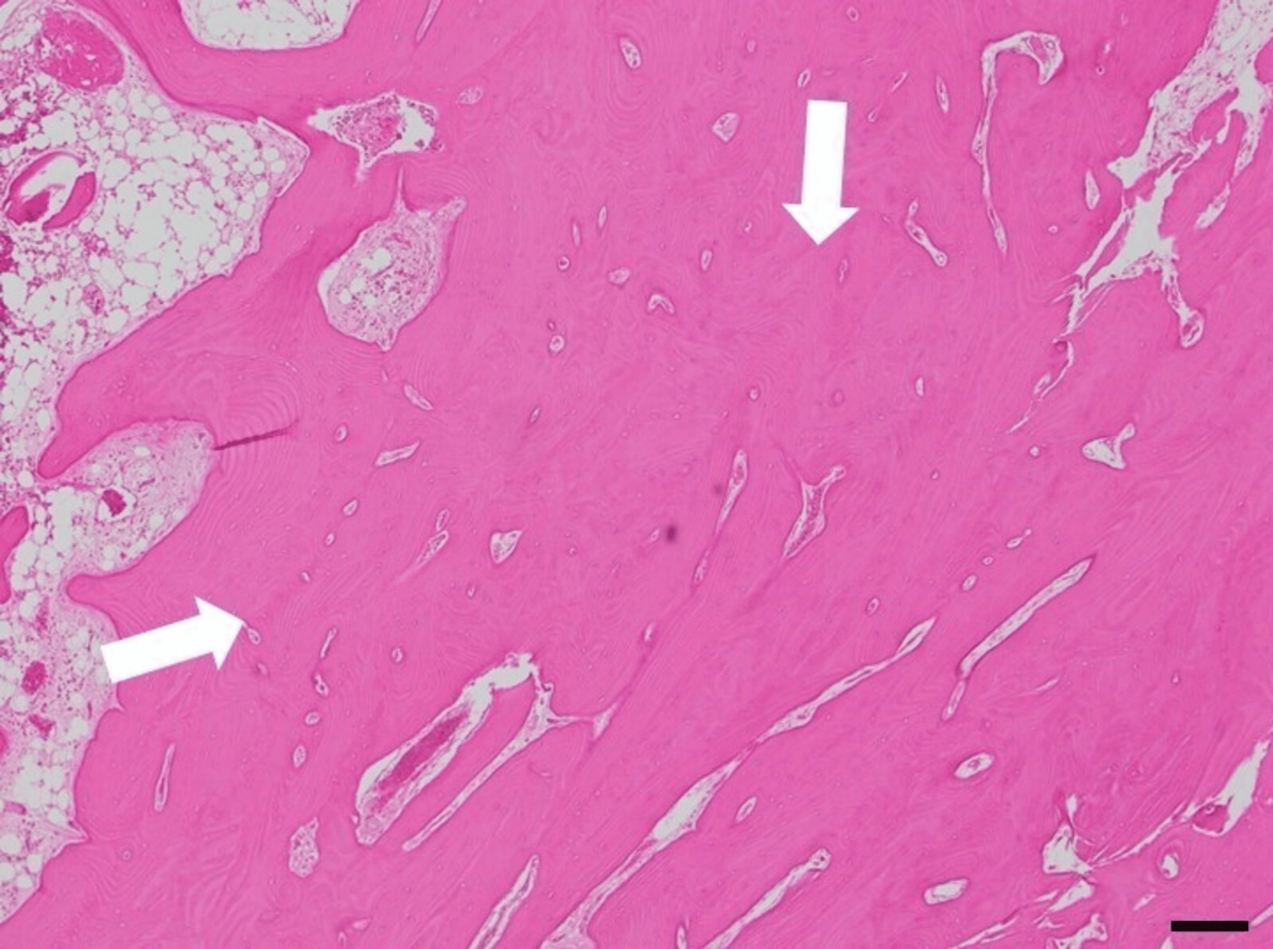
Histopathological images of the resected mass on hematoxylin and eosin staining. The appearance reveals an abnormal proliferation of thickened compact bone. Scale bars: 200 μm.

The surgical procedure was performed in the supine position under general anesthesia according to the following steps. A skin incision was made from the anterior-superior iliac spine to the midline of the rectus femoris muscle. The approach was made between the tensor fasciae latae and sartorius muscles, which revealed a bony mass beneath the rectus femoris (Figure 3A). As the quadriceps muscle attachments were interposed within the mass, they were detached from the lesion. Using a chisel, the mass was resected from its base, proceeding from distal to proximal. The mass was adherent to the femoral head and anterior joint capsule in the deep layer. The femoral head was visualized after resecting the mass along with a portion of the joint capsule (Figure 3B). Additional resection of the impinging bony protrusions was performed as much as possible (Figure 3C). Hip joint manipulation was then performed, which improved hip flexion to 60° and abduction to approximately 20° (Figure 3D). No hip instabilities were observed. Intraoperatively, we determined that a sufficient improvement in range of motion had been achieved. Therefore, lesions that were strongly fused with the host bone, including those at the anterior acetabulum and anterior femoral neck, were not intentionally resected. Histologically, the resected lesion consisted of compact bone tissue resembling cortical bone on hematoxylin and eosin staining (Figure 4).

**Figure 5:**
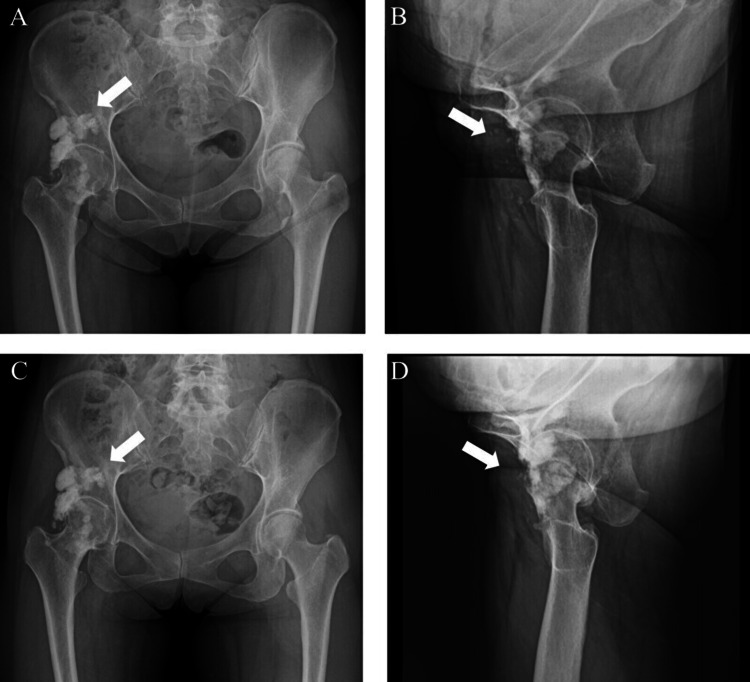
Postoperative plain radiographs of the right hip in anteroposterior position (A) and lateral position (B). Plain radiographs at last follow-up in anteroposterior position (C) and lateral position (D). No apparent regrowth was observed on the radiograph at the final follow-up.

At the last follow-up, 30 months after surgery, the hip joint range of motion showed mild limitations compared to immediately after surgery, with 45° of flexion and 25° of abduction. However, the patient continued to progress well, with no significant impact on daily activities. Radiographic evaluation showed no evidence of residual mass regrowth compared to immediate postoperative images (Figure 5).

## Discussion

In this article, we report a case in which surgical intervention led to improvements in joint range of motion and activities of daily living that were maintained 30 months postoperatively.

Although melorheostosis is benign, it can cause considerable clinical morbidity. It often begins without symptoms; however, a combination of bone sclerosis and soft tissue fibrosis can result in limb shortening, skeletal deformities, and joint stiffness. Over time, these changes may progress to severe pain and functional impairment in the affected limb [10]. The most frequently reported symptoms are pain (83.3%), deformity (54.1%), restricted range of motion (45.8%), numbness (37.5%), and muscle weakness (25.0%) [11].

In previous reports, symptom management was accomplished through orthopedic surgical interventions, pain medications, physical rehabilitation, and the use of bisphosphonates [12]. Surgical interventions for this condition encompass a wide range of procedures, including tendon lengthening, excision of fibrous and osseous tissue, fasciotomy, capsulotomy, various osteotomies, spinal decompression, nerve decompression, excision of hyperostosis, joint fusion, amputation, joint replacement, and limb lengthening techniques [13]. If conservative treatment is ineffective, surgery may be the only viable option; however, patients should be informed of the high risk of recurrence following invasive procedures [14]. Ruggiero et al. [15] reported a case of melorheostosis in which conservative treatment for restricted hip range of motion and pain failed to improve, leading to hip osteoplasty. Although the patient initially experienced symptom relief postoperatively, the symptoms recurred two months after surgery, and hip replacement surgery was proposed six months postoperatively [15].

In our case, the patient’s primary complaint was difficulty with daily activities caused by a limited range of motion; therefore, we performed mass resection and passive manipulation, which led to a favorable outcome. The extent of resection was determined intraoperatively through assessment of joint range of motion, in order to avoid impairing joint function by excising a mass not associated with the patient's symptoms. This patient showed a favorable outcome with no recurrence of symptoms at 30 months postoperatively; however, since the prognostic factors are still unclear and there is a possibility of regrowth of the residual mass, careful follow-up is necessary.

Although several hypotheses have been proposed, the exact cause of melorheostosis remains unclear. Several mechanisms have been postulated, such as developmental anomalies of the embryonic mesoderm involving the osseous and soft tissues, infections, and compromised vascular supply, resulting in defective intramembranous or endochondral bone formation [16]. Advances in molecular biology have revealed that mutations in the MAP2K1 gene are associated with the classic “dripping candle wax” pattern, while mutations in SMAD3 have been linked to the endosteal variant. Some cases of melorheostosis result from sporadic somatic mutations in MAP2K1, which encodes MEK1 protein kinase, a key component of the RAS/MAPK signaling pathway [17]. It is possible that the genetic heterogeneity of MAP2K1 results in phenotypic variability, beginning at the histopathologic level and ultimately manifesting in the clinical presentation [18]. Kang et al. reported that MAP2K1 mutations inhibit BMP2-mediated osteoblast mineralization and differentiation in vitro, which underlies the markedly increased osteoid observed in affected bone histology [19]. In the present case as well, it is possible that this genetic mutation contributed to enhanced bone formation, suggesting that genetic analysis may be necessary in future follow-up.

Owing to the rarity of diagnosed cases globally, evidence-based treatment guidelines have yet to be established. Therefore, management must be individualized for each patient and typically involves a multidisciplinary approach. There have been very few reports of partial resection and passive manipulation performed for melorheostosis involving the hip joint, and we believe that long-term follow-up in this case will provide valuable insights into the long-term outcomes. Therapeutic strategies should be guided by the symptom severity and the range of available treatment options [20].

## Conclusions

Melorheostosis is a rare benign bone disorder that can cause substantial functional impairment when associated with soft tissue involvement and joint impingement. In the present case, surgical resection of the impinging lesion combined with intraoperative joint mobilization resulted in significant improvement in hip range of motion and quality of life with no evidence of regrowth over a 30-month follow-up period. Given the rare and heterogeneous presentation of melorheostosis, individualized treatment strategies tailored to each patient’s symptoms and functional limitations are essential. Further clinical data are needed to establish standardized treatment guidelines.
